# Applying equity-centered principles in an interprofessional global health course: a mixed methods study

**DOI:** 10.1186/s12909-020-02141-1

**Published:** 2020-07-14

**Authors:** Lisa Stallwood, Prince A. Adu, Kate Tairyan, Barbara Astle, Annalee Yassi

**Affiliations:** 1 Faculty of Health Science, McMaster University, 1280 Main St W, Hamilton, ON L8S 4L8 Canada; 2 School of Population and Public Health, University of British Columbia, 2206 E Mall, Vancouver, BC V6T 1Z3 Canada; 3 British Columbia Centre for Disease Control, 655 W 12th Ave, Vancouver, BC V5Z 4R4 Canada; 4 Faculty of Health Sciences, Simon Fraser University, 8888 University Dr, Burnaby, BC V5A 1S6 Canada; 5 School of Nursing, Trinity Western University, 7600 Glover Rd, Langley, BC V2Y 1Y1 Canada

**Keywords:** Global health education, Curriculum development/evaluation, Values/attitudes, Professional development, Interprofessional education

## Abstract

**Background:**

Medical students, practitioners and other health professionals are commonly unprepared to address the many complex issues that emerge while conducting research in the Global South. As a response to identified deficiencies in global health education, a hybrid online/face-to-face multi-institutional credit course was developed based on the equity-centered principles advanced by the Canadian Coalition for Global Health Research (CCGHR), namely *Authentic partnering*, *Inclusion, Shared benefits*, *Commitment to the future*, *Responsiveness to causes of inequities*, and *Humility*. This study aimed to analyze the extent to which the course was effective in fortifying attitudes consistent with the CCGHR principles; identify successes and challenges; and assess how a course such as this can fill an identified gap.

**Methods:**

This interprofessional course was offered to 25 graduate and postgraduate students in various health professions and public health. Faculty were drawn from medicine, public health, nursing and social sciences from four universities in Western Canada. A pre-post retrospective survey, key informant interviews and participant observation were used to gather data for this study.

**Results:**

Findings showed that student attitudes regarding global health research and practice significantly evolved towards views consistent with the principles articulated. The multiple instructors and hybrid course format created both opportunities and challenges; the interprofessional nature of the cohort was considered a strong asset, as was the fact that many students came from the Global South. Some students suggested that the course could be further strengthened by concretely partnering with institutions in the Global South rather than offered solely to learners registered in universities in the Global North.

**Conclusions:**

While weaknesses were identified, results support the conclusion that a course focused on the CCGHR principles could be useful in preparing the next generation of global health researchers and practitioners to mitigate historical limitations in this field. Longitudinal follow-up is warranted to provide more definitive conclusions.

## Background

Greater awareness of present global health inequities has attracted increasing numbers of students and researchers from multiple disciplines to participate in global health initiatives [1]. However, complex ethical issues emerge when health researchers from high-income countries (HICs) engage in projects in low and middle-income countries (LMICs) [2, 3]. Without the appropriate training and experience for undertaking global health research, students may unintentionally harm or exploit already overburdened communities or individuals in low resource settings [4, 5]. Moreover, although global health may be seen in a positive light in the post-colonial world, it can sustain colonialist attitudes and behaviour through domination and unethical studies [6].

Numerous universities have taken up the challenge to teach global health skills to the next generation of researchers [7]. Some universities have begun to define competencies in global health education, often, however, without considering the needs and priorities of LMICs [8]. In response to such concerns, the Canadian Coalition for Global Health Research (CCGHR), a non-profit organization composed of practitioners, researchers and global health students, developed a set of research principles through a multiphase research process with extensive consultation in Canada and with global partners [9]. The principles, serving as an ethical paradigm, consist of six interrelated principles for equity centered research and practice: *Authentic partnering*, *Inclusion, Shared benefits*, *Commitment to the future*, *Responsiveness to causes of inequities*, and *Humility* [10]. While major funding agencies, including the Canadian Institutes for Health Research (CIHR) are supporting collaborations between Canadian researchers and researchers from LMICs, CIHR has acknowledged the importance for considering the CCGHR principles for equitable and ethical global health research partnerships [11].

In 2017, the British Columbia (BC) Chapter of the CCGHR hosted the inaugural BC Coalition Institute (BCCI-1) in Kelowna, BC to explore the application of these principles. Emerging from the positive feedback from this event, the institute organizers decided that it would be useful to design a course that embraced the principles of promoting health equity and equitable partnership development [1] that graduate students across Western Canada would be able to undertake as part of their course requirements. In contrast with current global health courses that focus on content learning, such as rigorous research methods and global health factual knowledge, the course aimed to be transformative - unpacking student values, beliefs and assumptions that may be remnants of colonialism, and instead promote values to align with the CCGHR principles.

This novel multi-institutional hybrid global health course was offered as a ‘for-credit’ elective course, mainly delivered online in the Summer 2019 session (May–June), through five synchronous online sessions, and included one three-day face-to-face weekend residency. Each week of the course was designated to focus on one of the following topics through readings, assignments and discussions: *introduction to the CCGHR principles; health equity; wicked problems; sustainable and equitable partnerships; ethical issues in global health*; and the *application of the CCGHR principles in the process of proposal development*. Students worked in teams to develop and present a proposal of a global health project that illustrated the use and application of the principles at the end of the course.

This graduate course was initially co-developed and co-lead by three professors (co-authors BA, KT and AY) from medical or nursing backgrounds and from three different universities in Western Canada; a PhD student (co-author PA) served as the teaching assistant. Global health practitioners from institutions in Western Canada and who teach Medicine, Nursing, Health Policy, and Population Health, attended the weekend residency and directly interacted with the students to share practical insights from their experiences in global health. This collaborative partnership embraced the diverse strengths of each team member and helped build capacity across the universities involved. The weekend residency provided an opportunity for face-to-face discussion about the topics taught in the course to that point, and to introduce and apply additional techniques such as logic framework analyses. The course was hosted in one of the partner institutions, using a pre-existing graduate-level credit course number which already had been approved as a hybrid (combined in-class and online) course in global health. The course was free to all graduate students registered at any university that was part of the Western Deans’ agreement which provided a tuition fee waiver for visiting students from partner institutions. The course partners have discussed the possibility of hosting the course every two years and alternating the hosting of the course between partner universities to assist with managing and sharing the workload. At this juncture, one of the partner institutions has hosted the course twice, as it ran consecutively and was more feasible to do so.

This study was conducted, first, to examine how the course influenced student values and attitudes in global health work; secondly, to identify the successes and challenges experienced by students and course instructors; and third, to explore the extent to which a course such as this could help fill a gap in current medical education with respect to the teaching of global health.

## Methods

There were 25 students enrolled in this course – the cap set for this initial launch. The students were at different stages in their educational careers, from a variety of educational disciplines and, although all were current students in universities in Western Canada, they came from various parts of the world, many from the Global South. Nineteen students were registered at the host university, with one or two from each of four other universities. Many students from the class had already established careers - including one as an engineer, one as a university instructor, four nurses, a veterinarian, five qualified physicians and three medical residents. The remainder of the class identified themselves simply as full-time students. Three data collection methods were used for this study: an online survey on attitudes with a pre-course questionnaire as well as a retrospective pre-post questionnaire; individual interviews of students and faculty of the course; and participant observation.

### Surveys

**Fig. 1:**
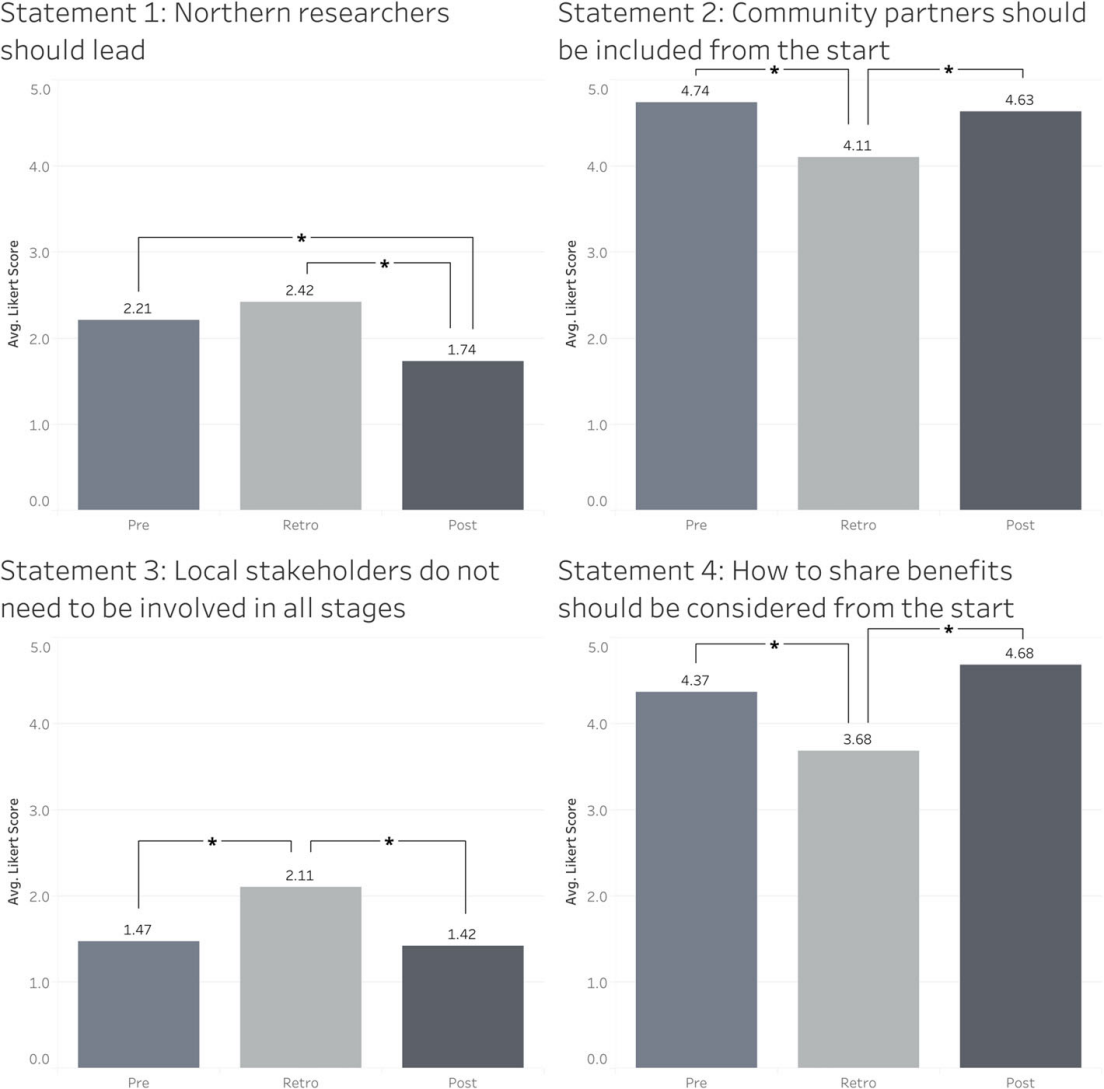
Comparing consistency of student responses with CCGHR principles pre-program, retrospectively pre-program, and post program. A paired t-test was used to determine significant differences between the average responses in the pre-course ratings, retrospective pre-course ratings and post course ratings (*n* = 19). Differences that are significant at the 95% level are indicated with an asterisk (*). See text under methods section for full statements

The online surveys were distributed at the beginning and end of the course with the aim of assessing changes in attitudes in global health research. The post-course survey was the same as the pre-course survey, with the addition of retrospective pre-post questionnaire items, asking students to rate their level of agreement with each statement as it was at the time of answering (post-participation in the course) and reflecting back as to their attitudes before the course, as has been done in other such studies [12]. The rationale for this technique is that respondents may have subjectively rated themselves a certain way prior to the course but after the completion of the course, they may reflect differently on what their previous attitudes towards global health research truly were. All 25 students completed the pre-course survey, and 24 students completed the post-course survey. Qualtrics (Qualtrics, Provo, UT) was used to collect and analyze the data. In addition to multiple-choice questions to characterize attitudes about various practices in global health, students were asked to record the extent to which they agreed with a set of statements grounded in the CCGHR principles on a 5-point Likert scale; 1-strongly disagree, 2-disagree, 3-not sure, 4-agree, 5-strongly agree. The average Likert score for each question was recorded. A paired t-test was used to determine significant differences between the average responses in the pre-course ratings, retrospective pre-course ratings and post course ratings (*n* = 19). The full statements are shown below, and abbreviated statements are presented in Fig. 1.

#### Statement 1

*In projects funded in the Global North, global health research/action is most efficient when researchers from the Global North take the lead in coordinating and designing the research or intervention, as they are usually the ones who write the proposals and are best trained to do this.*

#### Statement 2

*Global health research might best benefit all stakeholders if community partners, including those who are historically marginalized, are included in the research process from the very beginning.*

#### Statement 3

*Local stakeholders do not need to be involved in all parts of the research process such as developing research questions, determining data collections sites, and writing the final scientific paper, as they are often too busy.*

#### Statement 4

*When planning a research project, it is important to consider how benefits could be distributed amongst all partners involved in the research process, including partners who have not yet been involved.*

### Interviews

All students, co-instructors and guest faculty involved in the course were invited to a one-on-one semi-structured interview, conducted by lead author LS, who was not affiliated with the universities involved and not part of the teaching team. Nine students volunteered to participate as did all three lead course instructors, the PhD teaching assistant, and a senior faculty member and global health leader who participated as a facilitator in the face-to-face portion of the course. All interviews were audio-recorded, transcribed verbatim and analyzed using thematic analysis. NVivo 12 (QSR International Pty Ltd) was used to manage the data and categorize the text related to the priori and emergent themes.

### Participant observation

LS acted as a “participant observer” throughout the course itself, interacting with students and faculty as well as observing class discussions and instructor meetings. After the completion of the course, she consolidated all collected information to contextualize the data gathered through surveys and interviews. Observations offered by the co-authors were also incorporated, but, because of their positionality, care was taken to ensure that these observations did not influence the data reported herein.

## Results

### How the course influenced student attitudes towards global health research

Throughout the course, excitement and desire from students to continually discuss aspects of ethical global health research was observed through online discussion posts and during the face-to-face event. The results of the pre-course, retrospective pre-course and post-course ratings revealed that students had gained a greater appreciation of the principles of equitable global health research after the completion of the course. As demonstrated in the figure below (See Fig. 1), there was a significant difference between the average Likert scores in retrospective pre-course ratings and post-course ratings for all statements. The average scores in the surveys shifted in a direction that demonstrated an evolution of student attitudes towards global health work that more closely aligned with the CCGHR research principles. There was a significant difference between the pre-course ratings and retrospective pre-course ratings from all statements, except statement 1, demonstrating that students, in retrospect, characterized their previous attitudes, values and motivations related to global health research differently than they had done before they had taken the course.

When students were asked how researchers in the Global North can promote the participation of people who are historically marginalized in their research in the Global South, after the course, there was an 18.5% increase in the number of students who chose *to collaborate with their colleagues in the Global South to identify all relevant stakeholders*. Students had an increased awareness of the importance of the principle of inclusion, as illustrated by comments in the post-course survey noting that “*Colleagues in the global south would be familiar with the struggles of marginalized groups.”*

Prior to taking the course, when students were asked to select all partners who benefit from global health research, ~ 80% of the class chose *research participants* and *communities involved in research*; whereas, after the completion of the course, 95.8% of the class chose *research participants* and 100% of the class chose *communities involved in research*. Students evolved a greater appreciation to provide benefits to all individuals involved in the research process. One student wrote:

*“I think all partners benefit, and if research partners are not benefiting equally, then the research process should be revised to ensure properly shared benefits.”*

Students also demonstrated a greater understanding of the concept of humility and the ability to reduce subjectivity in qualitative research by creating dialogue between themselves and community participants on how they are positioned in their own research. The following quotes demonstrate student beliefs that acknowledge the importance of constant reflection after the completion of the course:

*“Dialogue between the researcher and their colleagues, supervisors, and community members is essential in ensuring that the researcher’s assumptions and beliefs don’t affect the research project.”*

*“Researchers in a position of power must always engage in self-reflection of where they stand in relation to the communities that they are engaging in.”*

A quarter of the class initially thought that global health research would be *unlikely* to provide harm to targeted communities. One student noted, “*although sustainability may be an issue, the work involved may be providing some kind of service to the communities for the duration of the project.”*

After the completion of the course, no students held the belief that service to LMICs is always positive and almost 70% of the class demonstrated awareness of the unforeseen harm that can indeed result from HIC activities in the Global South, regardless of good intentions. The following quotes from after the completion of the course exemplified this view:

*“Without the application of all principles, harm may be done unintentionally at the direct community or broader levels.”*

*“I think that even with good intentions, there may be a lot of unanticipated harm to the communities, and all researchers should be aware of this so that we know that it’s not enough to have good intentions.”*

Students absorbed concepts and elements from class discussions in a short duration of time and used these skills and evolved attitudes to prompt discussions with faculty and students and complete high-quality final projects. A faculty member stated:

*“It’s quite impressive to see in such a short period of time … [] ... students really absorbed so much of the topics that we wanted them to think about and read and discuss among themselves (Faculty 3).”*

After learning about the CCGHR principles, students commonly referred to them as a framework to guide future participation in global health research:

*“I think that the guidelines [principles] are a great lens to look at all research … []...not just research, but any partnership or recognizing any sort of relationship where there may be inequity and power … [] … not only global health relationships, but other areas of research, especially in Canada like Indigenous populations, or working with marginalized populations (student 6).”*

During the interviews, many students demonstrated an ability to critique research projects and research articles now that they are aware of the principles. Student 2 stated:

*“I’m actually very, very grateful because I can call on those principles to question and critically evaluate any proposed research project intervention.”*

Students would commonly reflect on their past experiences in global contexts and express how they wished they had taken this course and had their current knowledge before pursuing these initiatives in the past. Student 1 reflected on her previous work with a humanitarian medical non-governmental organization:

*“Were we actually widening inequalities with the programs we’re implementing or were we really thinking about sustainability? … []... If I were to go back and work with the organization, I’d want to be more involved in how these decisions are being made.”*

### Successes and challenges experienced by students and course instructors

The greatest perceived success was partnering across different universities to develop and deliver this unique course based on the CCGHR principles. This was a common view amongst all faculty members, and was illustrated by the following quote from faculty 1:

*“The first success is that we now have a credit course that’s based on the application of the principles of CCGHR. That in itself I think is a big success. I think the fact that we’re running it as a multi-institutional course itself is a success as well. So, both the fact -- the content innovation and the institutional innovation is a success, of course.”*

The majority of participants, including students and instructors, agreed that having three professors, one teaching assistant and other global health experts to facilitate the course was a positive feature. Students mentioned that this teaching style provided them with multiple perspectives, experiences and feedback relative to the global health principles. A student noted:

*“I think having the three instructors was amazing … []... it is better than having just the one professor, because I got to see so many examples and experiences, and that really spoke to like Global Health, having those different experiences and how every relationship is different (Student 6).”*

A faculty member demonstrated similar thoughts:

*“All the principles … []... are just the pivotal things to whether a Global Health project will be successful or not and have any impact that’s useful to the people for whom it supposedly created to serve. And it’s lifelong learning. All of us have to learn that it’s not something that you can read in a book. And so, the more you can interact with people about their experiences … []...I think, the better (Faculty 1).”*

There was a strong desire expressed from three students to have research partners from the Global South involved directly in the delivery of the course. Student 9 emphasized this view by stating:

*“I just wish some other perspectives were presented, especially perspectives from the Global South. We are in a* class*, a global health class, and everybody who is teaching us about global health are [based] local [ly].”*

Through observation, it was apparent that the course included many global perspectives from both instructors and students with vast experience and expertise on various topics of global health. Many of the discussions, class comments and presentations were initiated by students from the Global South or students who have worked in the Global South or with marginalized populations. The instructor team was composed of three women, two of whom are racialized and not “mainstream white”, and the teaching assistant was African - born and raised in Ghana. Nonetheless, another weakness of the course identified by some students was the lack of Indigenous perspective, recognized as increasingly important in developing an anti-colonial attitude in global health work. Despite the lack of Indigenous students or faculty, all faculty members mentioned the quality and diversity of the students in the class as an important positive feature of the course. A faculty member stated:

*“The diversity of students, I think it’s another hallmark of this course … []...we had this really mature and diverse group of students who were keen, who were enthusiastic about the course … [] … this was a dream group of students that I would imagine this course being taken by (Faculty 3).”*

Some challenges were faced due to the hybrid and multi-institutional nature of this course which precluded a lot of interaction for preparation within the instructor team. A faculty member explained that the course had to be developed and taught by …

*“Very busy people with their travel schedules, with their own teaching commitments and projects going on … []...I know how much effort it took to do that … relying on online communication presented some challenges, especially at the beginning, but then all our team members were able to adopt … []...to that way of working together (Faculty 3).”*

Two course leaders described the challenging nature to coordinate three instructors of a course, particularly as the instructors apparently did not know the content of each other’s presentations until they were given, had never discussed their views on various issues in global health and had not determined how adjustments would be made if, for unforeseen reasons, sessions went overtime. In terms of lessons learned, Faculty 1 said:

*“I think the first thing that I learned is the need to spend more time talking with colleagues about their visions and to not just assume that we’re all on the same wavelength and that we all have the same understanding of how things are going to flow.”*

### How the course could fill a gap in current global health education

The instructors of this course had taught other global health graduate courses and many of the students had taken other global health courses as well. All mentioned that previous courses they taught or took focused more on content and informational learning.

*“I think that we spend a lot of time in schools teaching knowledge, but not as much on transformative learning, attitudes and real-world problem solving. And I think especially in Global Health or for that matter working with indigenous communities, the principles are so important … []...Other courses I’ve taught have been much more focused on the content (Faculty 1).”*

This point was emphasized when another instructor described a graduate global health course that she taught,

*“Of course, I teach or incorporate the principles as part of the course that I’m teaching, absolutely, and I’m sure other Global Health instructors do … []... But, this course is of course different because the entire course was supposed to be built around the principles … [] … So, I think the course is unique from that perspective … []... they were the central piece of this course” (Faculty 3).*

The following quote from student 6 expressed this view:

*“I’d say that usually it’s [global health courses] 80% content and 20% application whereas this course was very group focused and a lot more on application. It had a lot less reading and a lot more doing which was a different way of doing the course, and a different way of learning.”*

When the student was asked whether any previous global health course that she has taken focused on attitudes for global health research, the response was:

*“No, no, no. They never brought in that. The first time I went for the BCCI event was when they brought in the aspect of value. Nobody ever talks about values, values of inclusion and … [] ...humility (student 6).”*

Student 5 commented that this course could be recommended because it “*brings out the fundamental questions that some people actually take for granted or may under-look.”*

In a description of the course’s value, an instructor said:

*“For us mentors to work with our students early on to understand that … []...yes, we have this privilege of having resources and coming from a place where knowledge is more accessible … []...we have to consider the concept of humility and shut up and listen more than talk. … [] … But unfortunately, many of us learn it through harsh lessons with broken partnerships and grants not continuing. That could be the reason why you are not having the type of trust and synergy with the communities that you are trying to work with. Yeah, so it’s very important for me that students understand those concepts [CCGHR principles] or at least think about them before engaging in global health work (Faculty 3).”*

## Discussion

Student interest in global health has grown over the last two decades, yet, despite the acknowledgement of the importance of ethical conduct [3, 13] respectful partnerships [9, 14], and the increasing appreciation that *attitudes* of health professionals undertaking research in LMICs is a better predictor of success than the professional’s *knowledge* or *skill set* [15], few courses are available that focus on principles for promoting equity in global health research. As such, courses such as this one merit attention to reorient global health education to focus not only on knowledge and technical skills but also on attitudes of aspiring global health professionals. Nonetheless careful scrutiny is needed as to how such courses are offered.

This course was developed to address a perceived gap in global health education by providing postgraduate medical students and other health professionals with an ethical framework to undertake equitable global health research. Students and instructors recounted this course as unique due to its focus on attitudes, values and practices of research rather than content learning, which is seen as a limitation of current global health education [8]. Through observation, surveys and interviews, it was evident that students formed stronger attitudes necessary to conduct ethical and equity-centered research. Not only did students acquire knowledge about ethical principles to which they previously may not have been introduced, but students were able to reflect on their evolved values and attitudes from the course and discuss how these novel attitudes will be pivotal in the future while undertaking global health research. To our knowledge, this is the only multi-institutional and interdisciplinary course that has been developed and implemented in Canada at a graduate level and focused specifically on student attitudes and practices rather than content learning. Our findings in this regard thus dovetail with those of Peluso and colleagues that global health education should focus on the attitudes and practices of students who will deal with marginalized populations in their fieldwork [16].

When comparing the pre-course ratings to the retrospective pre-course ratings, it became evident that students changed their understandings regarding appropriate attitudes for global health research from prior to taking the course; therefore, had we only compared the original pre-course ratings to post-course ratings, the data would not have exhibited the true extent to which students learned from the course. The retrospective pre-course score, based on a greater understanding of the issues involved, therefore seemed to be more accurate in assessing attitudes of the students before the course and revealed that students acquired new insight that enabled them to see limitations in their previous views and attitudes towards global health research that they had not seen at the start of the class. Our approach thus echoed the findings of others [17] on the value of a retrospective pre-post analysis.

Inter-institutional, interdisciplinary approaches and interprofessional collaboration have been identified as useful in delivering global health courses [16, 18], and interdisciplinary teaching has produced the type of changes in student knowledge, attitudes, beliefs and skills that align well with the purpose of this course [19]. However, some students and instructors alike commented on challenges attributable to having three professors leading the course, whose views were not perfectly aligned. Some participants in this course found the lack of clarity in leadership during the three-day workshop to be detrimental, while others liked the informality, the absence of the traditional hierarchy between instructors and students, and the multiplicity of views and approaches. Peluso and colleagues [16] noted that differences amongst faculty sometimes raise tensions and emphasized the importance of clarifying roles and responsibilities of each faculty member involved. The diversity of the cohort of students in this course was another important feature. Students from many professions, places of origin and varying stages in their educational and professional careers mimicked the type of collaboration that often exists in the interdisciplinary field of global health. Adams and colleagues [1] acknowledged the challenge of working in interdisciplinary teams with varying perspectives that do not always align, but the authors noted the importance for evolving global health professionals to adopt skills and experience that will allow them to effectively work in these environments.

A limitation identified in the course was the recognition of the lack of integration of Indigenous knowledge and concrete participation from First Nations communities. Instructors committed to ensuring that future iterations of the course would have a better representation of Indigenous perspectives, with at least one session devoted specifically to coloniality both in the Global North and Global South, and at minimum of one Indigenous guest speaker. Another limitation noted was that although the students and instructors had diverse personal and professional backgrounds and were involved in or lead multiple global health initiatives, they were all currently living in the Global North. Other universities have made better use of online learning modalities to create direct partnering between North and South and sometimes across institutions in multiple countries. For example, McMaster University’s Master of Science in Global Health program is delivered through a model in which universities from several continents offer joint learning. Such programs underline the perspective that global health is not about the Global North working with the Global South to address problems in the Global South, but rather facilitates a larger global perspective on our interconnected world and its unequal implications for health equity. Perhaps future offerings of this course can expand in this regard.

Importantly, this course not only emerged from the BC Coalition for Global Health, that runs a summer institute every second year, but students in the course are supported to participate in the summer institute, presenting the projects they developed in class. This helps solidify the involvement of trainees in a community-of-practice of global health practitioners and researchers, which itself may serve to encourage application of the principles, further preparing the next generation of global health researchers. Naidoo and Vernillo illuminated the value of a community of practice, a group of individuals with a common interest and desire to contribute to improving health ethics and strengthen the broader community through interdisciplinary collaboration [20].

## Conclusion

Global health education has been slow to provide researchers and practitioners with the necessary attitudes to address common issues that arise while undertaking global health work. This study showed a clear evolution of students’ views regarding global health research and practice, with evidence indicating that students acquired an increased understanding and appreciation of the CCGHR principles. With proper preparation and strategies to address challenges related to the multi-instructor, multi-dimensionality of the course, and ensuring a stronger Indigenous presence, concerns could be mitigated to ensure continued success. While longitudinal follow-up would be useful to ascertain the extent of application of the CCGHR principles in field settings, and direct involvement of institutions from the Global South could be advantageous, we argue that a course focusing on changing attitudes, and contributing to building a community-of-practice, is worth considering to promote the qualities widely believed to be of value in promoting global health.

## Data Availability

The datasets used and/or analyzed during the current study are available from the corresponding author on reasonable request.

## Abbreviations

HICs: High-income countries
LMICs: Low and middle-income countries
CCGHR: Canadian Coalition for Global Health Research
CIHR: Canadian Institute for Health Research
BC: British Columbia
BCCI-1: BC Coalition Institute
